# The Evolving Landscape of Hypertrophic Cardiomyopathy: Clinical Assessment, Precision Therapies, and Emerging Strategies

**DOI:** 10.3390/jcm15218498

**Published:** 2026-11-01

**Authors:** Alessandro Parodi, Beatrice Baù, Giorgio Piccolboni, Nicola Morat, Laura De Michieli, Marika Martini, Kalliopi Pilichou, Alberto Cipriani, Barbara Bauce, Chiara Calore

**Affiliations:** Department of Cardiac, Thoracic, Vascular Sciences and Public Health, University of Padova, Via Giustiniani 2, 35100 Padua, Italy

**Keywords:** hypertrophic cardiomyopathy, cardiac myosin inhibitors, left ventricular outflow tract obstruction, multimodality imaging, septal reduction therapy, precision medicine, gene therapy

## Abstract

Hypertrophic cardiomyopathy (HCM) is a genetically and phenotypically heterogeneous disease in which management requires integration of clinical assessment, cardiovascular physiology, multimodality imaging, functional evaluation, and arrhythmic risk. This review examines contemporary evaluation, risk stratification, and treatment of sarcomeric HCM across obstructive and non-obstructive phenotypes. In obstructive HCM, conventional negative inotropic therapy and septal reduction therapy remain established treatments, while cardiac myosin inhibitors have introduced a mechanism-based approach that improves left ventricular outflow tract obstruction, symptoms, and exercise capacity. Their role relative to septal reduction therapy should be individualized according to anatomy, hemodynamics, treatment burden, durability, and patient preference. In non-obstructive HCM, treatment remains largely symptom-directed, although recent trials of myosin inhibition have yielded divergent results, emphasizing the importance of appropriate patient selection. Emerging strategies targeting myocardial energetics and earlier disease stages may further expand treatment options. Gene-based therapies, including first-in-human *MYBPC3* replacement, offer the possibility of targeting the underlying molecular defect but require careful evaluation of safety, durability, and timing. Overall, HCM remains a paradigmatic disease for physiology-driven, phenotype- and stage-specific care, increasingly complemented by molecularly targeted therapies.

## 1. Introduction

Hypertrophic cardiomyopathy (HCM) is one of the most common inherited cardiac diseases, with an estimated prevalence of approximately 1:500 in the general population, although its true burden is likely higher due to underdiagnosis [1]. In adults, HCM is defined by a maximal end-diastolic left ventricular (LV) wall thickness ≥15 mm that is not solely explained by loading conditions or another disease capable of producing the observed degree of hypertrophy; a wall thickness of 13–14 mm may support the diagnosis in the presence of a positive family history or a pathogenic or likely pathogenic (P/LP) HCM-associated genetic variant [2,3]. However, HCM is genetically heterogeneous: it is most commonly inherited as an autosomal dominant trait, but alternative inheritance mechanisms are possible, while common genetic modifiers and non-Mendelian mechanisms may contribute to altered disease expression [4,5,6]. Traditionally, a P/LP variant is identified in approximately 30–60% of clinically diagnosed patients, with *MYBPC3* and MYH7 accounting for most genotype-positive cases [4,5,6]. Recently, a fundamental gene curation analysis has refined the current knowledge about the genetic architecture of HCM, confirming a restricted set of genes with robust disease associations [6]. Genetic testing therefore has an established role in diagnostic clarification and cascade family screening, whereas its contribution to individual prognostic stratification remains more limited [2,3]. Several infiltrative, metabolic, syndromic, and neuromuscular disorders may produce an HCM-like phenotype but differ substantially in pathophysiology, natural history, and treatment. Their recognition remains essential during diagnostic evaluation; however, these phenocopies are beyond the scope of the present review [2,3]. Contemporary HCM care increasingly integrates clinical phenotype, multimodality imaging, functional assessment, arrhythmic risk, and genetic information. At the same time, cardiac myosin inhibitors have introduced sarcomere-directed therapy for obstructive HCM, while advances in non-obstructive disease, heart failure management, and gene-based approaches are broadening the therapeutic landscape [2,3,5]. This review summarizes current strategies for evaluation, risk stratification, and treatment across the HCM spectrum, with particular attention to phenotype-directed management and emerging disease-modifying therapies.

## 2. Clinical Evaluation and Multimodality Imaging

### 2.1. Clinical Assessment and Electrocardiography

Clinical assessment remains central to the initial evaluation and longitudinal follow-up of patients with HCM. A detailed history should characterize exertional dyspnea, chest pain, palpitations, presyncope or syncope, and exercise intolerance, together with their temporal evolution and impact on functional capacity. Particular attention should be given to unexplained syncope, previous atrial or ventricular arrhythmias, and a family history of HCM, premature sudden cardiac death (SCD), or heart failure. Assessment of concomitant hypertension, coronary artery disease, obesity, sleep-disordered breathing, and other comorbidities is also relevant, as these may influence symptoms, ventricular loading conditions, and disease progression [2,3]. Physical examination may provide important clues, particularly in obstructive disease. The characteristic systolic ejection murmur related to dynamic left ventricular outflow tract obstruction (LVOTO) typically increases with maneuvers that reduce preload, such as standing or the Valsalva maneuver, and decreases with maneuvers that increase venous return or afterload, such as squatting. However, physical findings may be subtle or absent, particularly in patients without resting obstruction, and cannot reliably define disease severity [2,3]. The resting 12-lead electrocardiogram (ECG) is an essential component of both initial assessment and follow-up. It is abnormal in approximately 90–95% of patients with an overt HCM phenotype, although no single electrocardiographic pattern is diagnostic [2,7,8]. Common abnormalities include increased QRS voltages, repolarization changes with ST-segment depression and T-wave inversion, pathological Q waves, left atrial abnormalities, and intraventricular conduction disturbances. Deep T-wave inversion, particularly in the precordial and inferolateral leads, is frequently associated with apical hypertrophy, whereas pathological Q waves and diffuse ST–T abnormalities are less phenotype-specific and may occur across different HCM morphologies [7,8]. Importantly, ECG voltage correlates only weakly with the magnitude or distribution of hypertrophy, and marked structural disease may occur without conventional voltage criteria for LV hypertrophy [7]. Electrical abnormalities may precede overt hypertrophy, particularly in genotype-positive relatives undergoing family screening. Conversely, a normal ECG is uncommon but does not exclude HCM [3,7,8]. The ECG may also reveal findings that should prompt consideration of alternative causes of LV hypertrophy, including ventricular pre-excitation, conduction disease, or unexpectedly low QRS voltages in relation to wall thickness [3,7]. Ambulatory ECG monitoring is recommended at initial evaluation and every 1–2 years to detect NSVT, AF, and other arrhythmias, with extended monitoring for symptoms or suspected paroxysmal AF [2,3]. Although several ECG-derived markers have been associated with adverse outcomes, surface ECG findings alone are insufficient for risk stratification and should be integrated with clinical, imaging, and arrhythmic data [2,3,8].

### 2.2. Echocardiography

Transthoracic echocardiography is the first-line imaging modality in HCM, providing assessment of LV morphology, systolic and diastolic function, left atrial remodeling, mitral valve anatomy, and dynamic obstruction [2,3,9,10]. Maximal LV wall thickness should be measured systematically across myocardial segments, with careful avoidance of oblique sections or inclusion of right ventricular structures. Although asymmetric septal hypertrophy is the most common pattern, apical, mid-ventricular, concentric, and other morphological variants should be recognized. Contrast echocardiography may improve endocardial definition when apical morphology is suboptimally visualized, whereas CMR is preferred when wall thickness or phenotype remain uncertain [9,10]. LV ejection fraction is usually preserved or supranormal and may remain insensitive to early systolic impairment. Global longitudinal strain can detect subclinical contractile abnormalities, although its incremental role in routine clinical decision-making remains incompletely established [9]. Diastolic dysfunction is frequent and reflects impaired relaxation and increased myocardial stiffness. Its assessment requires integration of mitral inflow, tissue Doppler velocities, left atrial volume and function, including left atrial strain, and pulmonary pressures, as no single echocardiographic parameter reliably estimates LV filling pressures in HCM [9,10]. Assessment of LV outflow tract obstruction (LVOTO) is a central component of echocardiography in HCM. Obstruction is defined by a peak instantaneous LVOT gradient ≥30 mmHg at rest or during physiological provocation, while a gradient ≥50 mmHg identifies the conventional hemodynamic threshold for obstruction-directed therapy in symptomatic patients [2,3]. Because LVOTO is dynamic, Valsalva and standing maneuvers should be performed when the resting gradient is <50 mmHg. In symptomatic patients without significant resting or provocable obstruction, exercise echocardiography is recommended to identify latent LVOTO under physiological conditions [2,3,9]. Dynamic obstruction most commonly results from the interaction between septal hypertrophy and systolic anterior motion (SAM) of the mitral valve, but abnormalities of leaflet length, chordae, and papillary muscle position may contribute substantially to its mechanism [9,10]. SAM-related mitral regurgitation is typically posteriorly directed, whereas atypical jet direction or disproportionate regurgitation should raise suspicion of intrinsic mitral valve disease. Defining these mechanisms is particularly relevant when septal reduction therapy is considered [9,10]. Echocardiography also identifies mid-ventricular obstruction, apical aneurysms, progressive atrial enlargement, pulmonary hypertension, and transition toward LV systolic dysfunction. Serial assessment allows tracking phenotypic and hemodynamic evolution; in clinically stable patients, repeat echocardiography every 1–2 years is generally recommended, with earlier reassessment after changes in symptoms or clinical status [2,3].

### 2.3. Cardiac Magnetic Resonance

Cardiac magnetic resonance (CMR) provides complementary structural and tissue characterization and has become an integral component of contemporary HCM assessment [2,3,9]. Its high spatial resolution allows accurate quantification of LV volumes, mass, ejection fraction, and maximal wall thickness, particularly when echocardiographic quality is poor. CMR is particularly valuable for identifying apical and focal hypertrophy, mid-ventricular disease, apical aneurysms, and abnormalities of the mitral valve and papillary muscles, while also helping distinguish HCM from alternative causes of LV hypertrophy [2,3,9,10]. Late gadolinium enhancement (LGE) identifies focal replacement fibrosis and is present in a substantial proportion of patients with established HCM. Its distribution is typically patchy and non-ischemic, frequently involving hypertrophied segments and right ventricular insertion points. Beyond diagnostic characterization, increasing LGE burden is associated with ventricular arrhythmias, sudden cardiac death (SCD), heart failure progression, and adverse ventricular remodeling [11,12]. Extensive LGE, conventionally defined as ≥15% of LV mass, is currently used as a modifier of SCD risk when primary-prevention ICD decisions remain uncertain [2,3,11]. However, fibrosis-related risk appears continuous rather than confined to a single threshold, and recent prospective data from the NHLBI HCM Registry further support the prognostic relevance of quantitative scar burden [12]. CMR also provides sensitive detection of features associated with adverse outcomes, including LV apical aneurysm and progression toward systolic dysfunction. These findings have direct implications for risk stratification and may be missed or underestimated by echocardiography [2,3]. In obstructive HCM, CMR can additionally clarify the anatomical contributors to LVOTO, including septal morphology and abnormalities of the mitral-papillary muscle apparatus, and may support planning of septal reduction therapy when echocardiographic anatomy is insufficiently defined [2,9,10]. Native T1 mapping and extracellular volume (ECV) quantification extend tissue characterization beyond focal replacement fibrosis by providing markers of diffuse interstitial myocardial abnormalities [9,13]. Increased native T1 and ECV have been associated with greater disease burden and adverse remodeling, including in myocardial regions without overt LGE. Their incremental prognostic value and optimal thresholds, however, remain insufficiently established for routine therapeutic or ICD decision-making [9,13]. CMR therefore complements echocardiography, integrating precise phenotypic definition with myocardial tissue characterization. Repeat CMR may be considered when clinical status changes or new risk markers emerge and, in otherwise stable patients, periodic reassessment every 3–5 years may be useful to detect progression of fibrosis, changes in ventricular function, or development of high-risk features [2,9].

### 2.4. Cardiac Computed Tomography

Cardiac computed tomography (CCT) has a complementary role in HCM, particularly when CMR is unavailable or contraindicated and when specific anatomical or coronary questions need to be addressed [2,3,9,14]. Its high spatial resolution permits accurate assessment of LV morphology and wall thickness and may further characterize the mitral-subvalvular apparatus in obstructive HCM, including when septal reduction therapy is considered [9,14]. Coronary CT angiography (CCTA) is particularly valuable in patients with angina, allowing non-invasive exclusion of concomitant obstructive epicardial coronary artery disease [14,15]. This distinction is clinically relevant because myocardial ischemia in HCM frequently occurs despite the absence of significant epicardial stenoses and may reflect coronary microvascular dysfunction [15]. CCTA may also support procedural planning, particularly before alcohol septal ablation, by defining septal perforator anatomy [14,16]. Late iodine enhancement (LIE) extends CCT beyond anatomical and coronary assessment by providing myocardial tissue characterization analogous to LGE-CMR. HCM-specific studies have shown that LIE-CT can identify focal myocardial scar and estimate fibrosis burden with reasonable agreement with CMR [17,18,19]. Conventional LIE-CT remains limited by lower contrast-to-noise ratio, radiation exposure, iodinated contrast administration, and reduced sensitivity for small non-ischaemic scars [14,20,21]. Nevertheless, it may represent a useful alternative when tissue characterization is clinically relevant and CMR cannot be performed [14,20,21]. Importantly, ongoing advances in spectral and photon-counting CT are expanding myocardial tissue characterization and may progressively narrow the gap with CMR by integrating coronary anatomy, cardiac morphology, function, and tissue characterization within a single examination [20]. CMR currently remains the reference technique for myocardial fibrosis assessment, but the role of CCT in HCM is likely to expand substantially in the near future.

### 2.5. Exercise Testing and Cardiopulmonary Exercise Testing

Exercise testing provides objective assessment of functional capacity and dynamic cardiovascular physiology in HCM. Conventional testing evaluates workload, symptoms, heart rate and blood-pressure responses, and exercise-induced arrhythmias, while exercise echocardiography is particularly useful for detecting latent LVOTO when resting or bedside-provoked gradients are not significant [2,3]. Exercise testing is also reasonable when objective functional or prognostic assessment is required [2]. Cardiopulmonary exercise testing (CPET) extends this evaluation by integrating gas exchange with cardiovascular performance. Key variables include peak oxygen uptake (peak VO_2_), VE/VCO_2_ slope, ventilatory threshold, oxygen pulse, and chronotropic and blood-pressure responses [22,23,24]. Exercise limitation in HCM is multifactorial and may reflect impaired stroke-volume and cardiac-output reserve, diastolic dysfunction, LVOTO, mitral regurgitation, chronotropic incompetence, and abnormal pulmonary vascular responses [25,26]. Combined CPET and non-invasive hemodynamic assessment have shown peak cardiac output to be a major determinant of exercise capacity, while impaired ventilatory efficiency relates to greater diastolic burden [25]. CPET also provides prognostic information. Reduced peak VO_2_ and increased VE/VCO_2_ slope are associated with heart failure progression, transplantation, and mortality [23,24,25,26,27], while exercise-derived variables have also been linked to arrhythmic outcomes [28]. Their role in SCD stratification remains complementary, as neither CPET parameters nor abnormal exercise blood-pressure response constitute stand-alone indications for primary-prevention ICD implantation [3]. CPET is particularly useful when symptoms appear disproportionate to resting disease severity or functional class is uncertain. Exercise capacity may provide prognostic information beyond resting hemodynamics and exercise-induced LVOT gradient [29]. In advanced non-obstructive HCM and heart failure, CPET also supports assessment for advanced therapies, including heart transplantation [2]. Serial CPET may therefore complement clinical and imaging follow-up by objectively tracking functional status and treatment response.

## 3. Clinical Phenotypes and Risk Stratification

### 3.1. Obstructive HCM

Left ventricular outflow tract obstruction (LVOTO) is a major determinant of the clinical phenotype in HCM. Broadly, resting obstruction is present in approximately one third of patients; a further third develop LVOTO only with physiological provocation, particularly exercise, while the remaining third remain non-obstructive despite provocation [2,3]. LVOTO is a dynamic phenomenon resulting from the interaction between ventricular geometry, myocardial hypercontractility, loading conditions, and abnormalities of the mitral–subvalvular apparatus. In most patients, systolic anterior motion of the mitral valve brings the leaflets toward the septum, producing both dynamic outflow obstruction and, frequently, posteriorly directed mitral regurgitation [9]. The hemodynamic consequences extend beyond the instantaneous gradient and include reduced forward stroke volume, increased intracavitary and LV filling pressures, secondary mitral regurgitation, and left atrial pressure overload. Accordingly, obstructive HCM is frequently associated with exertional dyspnea, chest pain, exercise intolerance, presyncope or syncope, although substantial gradients may also be present in minimally symptomatic patients [2,3]. The relationship between LVOTO and outcome is more complex than its well-established association with symptoms. In a landmark cohort of 1101 patients, resting obstruction was independently associated with progression to severe heart failure symptoms and HCM-related mortality [30]. Subsequent studies showed that its prognostic impact is strongly influenced by the broader clinical phenotype, particularly symptom burden, with functional limitation becoming a dominant determinant of outcome once advanced symptoms develop [31]. Importantly, risk does not appear to increase linearly with progressively higher gradients above the diagnostic threshold, supporting the concept that LVOTO should not be interpreted as an isolated continuous measure of disease severity [30]. Comparisons between obstructive and non-obstructive HCM have subsequently provided a more nuanced picture. A meta-analysis including 7731 patients found no significant difference in long-term HCM-related mortality between the two phenotypes [32]. More recently, a systematic review of 61 observational studies including more than 52,000 patients similarly found no significant difference in the relative risk of all-cause, HCM-related, or heart failure-related mortality, although the relative risk of sudden cardiac death was higher in obstructive HCM [33]. These findings reinforce the concept that obstruction represents an important component of disease burden but does not alone define long-term prognosis. Myocardial fibrosis, hypertrophy burden, diastolic dysfunction, atrial remodeling, ventricular dysfunction, arrhythmic substrate, and associated morphological abnormalities remain important contributors to outcome [2,3]. Mid-ventricular obstruction represents a less common clinically distinct obstructive phenotype, resulting from systolic apposition within the mid-LV cavity rather than classical SAM-mediated subaortic obstruction. It is closely associated with apical pressure overload and apical aneurysm formation and has been linked to greater symptom burden, progressive heart failure, and ventricular arrhythmic events [34,35]. Importantly, mid-ventricular obstruction may not always generate a conventionally measurable high Doppler gradient. In a multicenter cohort of patients with HCM and apical aneurysms, 95% demonstrated mid-LV obstruction with complete systolic emptying, whereas only 19% had a Doppler systolic gradient ≥30 mmHg, suggesting that recognition of this phenotype requires integration of cavity morphology and systolic flow rather than reliance on gradient magnitude alone [35]. Thus, LVOTO is best regarded as a dynamic hemodynamic expression of the broader HCM phenotype, with important implications for symptoms, functional limitation, treatment selection, and overall clinical assessment [2,3].

### 3.2. Non-Obstructive HCM

Non-obstructive HCM comprises patients in whom a significant LVOT gradient is absent both at rest and during physiological provocation. In contrast to obstructive disease, symptoms result from several interacting mechanisms, including impaired ventricular relaxation and compliance, elevated filling pressures, reduced stroke-volume reserve, microvascular dysfunction, myocardial ischemia, chronotropic limitation, and atrial or ventricular arrhythmias [2,3]. Accordingly, exercise intolerance, dyspnea, and chest discomfort may be substantial despite preserved LVEF and the absence of LVOTO. The natural history of non-obstructive HCM is heterogeneous. In a cohort of 706 patients followed for a median of 5 years, overall survival was similar to that expected in an age- and sex-matched population, suggesting a relatively favorable course in many patients [36]. However, a subset develops progressive ventricular remodeling and heart failure, emphasizing that the absence of obstruction should not be interpreted as a sign of mild disease or benign long-term prognosis [36]. Progressive heart failure in non-obstructive HCM may occur through distinct pathways. In a longitudinal clinicopathological study, almost half of patients developing severe progressive HF had non-obstructive disease with preserved systolic function, whereas approximately one third progressed with LV systolic dysfunction [37]. Advanced HF with preserved LVEF is characterized predominantly by severe diastolic dysfunction, restrictive physiology, elevated filling pressures, atrial remodeling, and impaired cardiac-output reserve. Importantly, this phenotype may progress to refractory symptoms and require heart transplantation despite a nondilated LV and preserved global systolic function [38]. Reliance on LVEF alone may therefore substantially underestimate disease severity. A distinct adverse trajectory is the development of HCM with LV systolic dysfunction (HCM-LVSD), conventionally defined by LVEF < 50% [39]. The historical terms “burned-out” or “end-stage” HCM are imprecise because systolic dysfunction does not invariably represent a terminal disease phase and its clinical course is highly heterogeneous. In the SHaRe Registry, HCM-LVSD occurred in approximately 8% of patients and was associated with an increased risk of death, cardiac transplantation, or LV assist-device implantation, although progression varied substantially among individuals [39]. Atrial fibrillation, greater myocardial fibrosis, lower LVEF, and sarcomeric genotype were associated with development or adverse outcomes of HCM-LVSD [39]. Earlier longitudinal studies described a dilated-hypokinetic pattern of progression, in which systolic dysfunction was accompanied by LV cavity enlargement, progressive wall thinning, and adverse remodeling, with a particularly unfavorable prognosis [39]. However, systolic dysfunction in HCM may also occur without substantial ventricular dilatation, and contemporary terminology should therefore describe ventricular function and remodeling separately rather than considering all patients with LVEF <50% as having a uniform “dilated” or terminal phenotype [39,40]. Contemporary cohorts further suggest that prognosis after development of LV systolic dysfunction is variable and has improved with modern HF, device, and advanced therapies [41]. Non-obstructive HCM should therefore be regarded as a heterogeneous phenotype rather than simply the absence of LVOTO. Longitudinal assessment should focus on the evolution of symptoms, exercise capacity, diastolic function, atrial remodeling, myocardial fibrosis, arrhythmic burden, ventricular dimensions, and systolic function, with particular attention to patients developing either advanced HF with preserved LVEF or HCM-LVSD. These trajectories, rather than obstruction status alone, ultimately determine progression toward advanced heart failure and the need for advanced therapies [2,3].

### 3.3. Sudden Cardiac Death Risk Stratification

Sudden cardiac death (SCD) remains one of the major preventable complications of HCM, although its incidence in contemporary cohorts is low. Risk is heterogeneous and evolves over time; therefore, systematic reassessment is recommended at diagnosis and every 1–2 years, integrating clinical history, ambulatory ECG, echocardiography, and CMR [2,3]. Patients with previous cardiac arrest or hemodynamically significant sustained ventricular arrhythmias are considered at high risk for recurrence, whereas primary-prevention assessment requires integration of multiple markers. The European strategy is centered on the HCM Risk-SCD model, which estimates 5-year SCD risk from age, maximal LV wall thickness, left atrial diameter, LVOT gradient, family history of SCD, NSVT, and unexplained syncope [42]. The 2023 ESC guidelines classify estimated risk as low (<4%), intermediate (4–<6%), or high (≥6%), using additional disease features to adjust the calculated estimate [3]. Importantly, the model does not account for several contemporary risk markers such as LV systolic dysfunction, apical aneurysm, or myocardial fibrosis. The 2024 AHA/ACC approach places greater emphasis on additional risk markers. The 2024 AHA/ACC approach places greater emphasis on additional risk markers. Major risk factors include HCM-related SCD in a first-degree or close relative (≤50 years old), massive LV hypertrophy (≥30 mm), recent syncope considered likely arrhythmic, LV apical aneurysm with scar, and LVEF <50%. The presence of at least one major risk factor makes primary-prevention ICD implantation reasonable (Class IIa), whereas extensive LGE or NSVT may support ICD consideration (Class IIb) in patients without major risk factors when the decision remains uncertain [2,43]. NSVT is particularly relevant when episodes are frequent (e.g., ≥3 runs), longer (e.g., ≥10 beats), or faster (e.g., ≥200 bpm). Clinical interpretation remains essential: syncope should be distinguished from hemodynamic or neurocardiogenic mechanisms, while the prognostic significance of NSVT is influenced by its burden and characteristics rather than its presence alone. An LGE burden ≥15% of LV mass is conventionally considered extensive and represents an additional marker of SCD risk [11]. However, fibrosis-related risk appears continuous, and recent prospective HCM Registry data further support the prognostic value of quantitative scar burden alongside ventricular function and other risk markers [12]. LV apical aneurysm identifies another arrhythmogenic substrate that may not be adequately represented by conventional risk models [44]. Thus, contemporary SCD stratification should integrate major clinical features, arrhythmic burden, ventricular function, and myocardial substrate to support individualized shared decision-making regarding primary-prevention ICD therapy [2,3].

## 4. Management of Major Clinical Manifestations

### 4.1. Atrial Fibrillation and Thromboembolic Prevention

Atrial fibrillation (AF) is the most frequent sustained arrhythmia in HCM and is associated with worsening symptoms, heart failure progression, and thromboembolic events [45,46]. Its hemodynamic impact may be particularly relevant because loss of atrial contraction and rapid ventricular rates further impair LV filling in a disease characterized by reduced ventricular compliance. Left atrial enlargement, diastolic dysfunction, increasing age, and advanced functional limitation identify patients at greater risk of developing AF [2,3]. Thromboembolic risk in HCM with AF is substantially increased and is not adequately captured by the conventional CHA_2_DS_2_-VA score [45]. Accordingly, oral anticoagulation is recommended in all patients with clinical AF irrespective of conventional stroke-risk scores, with direct oral anticoagulants preferred over vitamin K antagonists in the absence of contraindications [2,3]. For device-detected subclinical AF, the 2024 AHA/ACC guideline recommends anticoagulation for episodes lasting >24 h and supports individualized consideration for episodes between 5 min and 24 h according to AF burden, additional thromboembolic risk factors, and bleeding risk [2]. Extended ambulatory monitoring is particularly relevant in patients at increased risk of AF because clinically silent episodes are common. Because AF may be poorly tolerated in HCM, maintenance of sinus rhythm is generally favored when feasible [2,3]. Electrical cardioversion and antiarrhythmic therapy can be used according to symptoms, ventricular function, comorbidities, and concomitant obstruction, whereas beta-blockers or non-dihydropyridine calcium channel blockers are preferred when a rate-control strategy is selected. Catheter ablation is an established option for symptomatic AF when pharmacological rhythm control is ineffective, although recurrence rates are higher than in patients without HCM [47]. Concomitant surgical AF ablation may be considered in selected patients undergoing septal myectomy [2]. Management of AF in HCM therefore requires early detection, prompt anticoagulation, and a rhythm-control strategy when feasible.

### 4.2. Ventricular Arrhythmias

Ventricular arrhythmias in HCM range from asymptomatic NSVT to sustained ventricular tachycardia (VT), ventricular fibrillation, and electrical storm. While NSVT primarily contributes to SCD risk stratification, sustained or recurrent ventricular arrhythmias require specific treatment aimed at terminating acute events and reducing subsequent arrhythmic burden [2,3]. Common reversible triggers, including electrolyte abnormalities, myocardial ischemia, and proarrhythmic drugs, should be identified and corrected whenever possible. Beta-blockers generally represent the initial pharmacological approach. In patients with symptomatic VT or recurrent appropriate ICD therapies despite beta-blockade, amiodarone, sotalol, mexiletine, or dofetilide may be used according to the arrhythmic substrate, ventricular function, comorbidities, and long-term toxicity [2]. Antiarrhythmic treatment reduces recurrent arrhythmias and ICD therapies but does not replace ICD protection when indicated. Catheter ablation is an important option for recurrent sustained monomorphic VT or repeated ICD therapies despite optimal pharmacological treatment [2,3]. The substrate may be intramural or epicardial, particularly in patients with extensive fibrosis, adverse LV remodeling, or apical aneurysms, and combined endocardial–epicardial approaches may therefore be required [44]. In a meta-analysis of six studies including patients with drug-refractory HCM, VT ablation achieved high acute procedural success and approximately 70% freedom from recurrent VT after the index procedure [48]. Electrical storm requires immediate correction of precipitating factors, antiarrhythmic therapy administration and reduction in sympathetic activation; catheter ablation and autonomic modulation or mechanical circulatory support should be considered at experienced centers [3].

### 4.3. Device Therapy: ICD, Pacing and Cardiac Resynchronization Therapy

Device therapy in HCM should be tailored to the patient profile, considering his arrhythmic, conduction, and heart failure phenotype. When an indication for ICD implantation has been established, selection between transvenous and non-transvenous systems should consider age, need for bradycardia or antitachycardia pacing, vascular access, and long-term lead-related complications [2,3]. Subcutaneous ICDs avoid intravascular leads but cannot provide pacing or conventional ATP, whereas transvenous systems retain these capabilities at the cost of possible long-term lead-related complications. Emerging extravascular systems are beginning to challenge this traditional dichotomy. The substernal extravascular ICD (EV-ICD) preserves the vascular space while providing defibrillation and ATP; in a recent analysis of the pivotal EV-ICD study including 41 patients with HCM, implantation and defibrillation success were high and major complication rates were comparable with those observed in patients without HCM [49]. Fully leadless modular approaches combining an S-ICD with a communicating leadless pacemaker capable of ATP have also demonstrated favorable early performance and may further expand non-transvenous device strategies, although HCM-specific experience remains limited [50]. In previous studies, dual-chamber RV pacing showed the ability to reduce LVOT gradients; however, randomized studies have shown inconsistent long-term symptomatic benefit, and pacing should not be considered an alternative to contemporary pharmacological treatment or septal reduction therapy for obstructive HCM [2,3]; thus, permanent pacing should primarily follow conventional indications in HCM. CRT has a more specific role in patients with HCM-LVSD and electrical dyssynchrony. Conventional CRT indications apply to patients with marked systolic dysfunction and LBBB, while the 2024 AHA/ACC guideline also considers CRT reasonable for symptom reduction in selected patients with non-obstructive HCM, LVEF <50%, LBBB, and persistent NYHA class II–IV symptoms who require an ICD [2]. However, HCM-specific evidence remains limited to small observational series, although improvement in functional class, LVEF, and reverse remodeling has been reported in selected patients [51].

### 4.4. Heart Failure Management

Heart failure (HF) in HCM is heterogeneous and management should be driven by the leading mechanism of functional limitation, including LVOTO, diastolic dysfunction, mitral regurgitation, atrial arrhythmias, or progressive systolic dysfunction [2,3]. In patients with congestion and no significant obstruction, cautious use of diuretics may be useful, but excessive preload reduction should be avoided in those with small LV cavities or restrictive physiology. Importantly, symptomatic HCM with preserved LVEF should not be considered equivalent to conventional HFpEF, and evidence supporting routine application of contemporary HFpEF pharmacotherapy to HCM remains limited [2,52]. Development of LVEF <50% defines HCM-LVSD and represents a clinically relevant transition associated with adverse outcomes [2,39,41]. Guideline-directed therapy for HF with reduced EF should be initiated if tolerated, while potentially reversible contributors including coronary artery disease, valvular disease, persistent tachyarrhythmias, and treatment-related myocardial depression should be investigated [2,52]. Generally, it should be kept in mind that evidence for conventional HF therapy in HCM-LVSD is extrapolated from the general HF population because patients with HCM have been poorly represented in pivotal randomized trials. Advanced HF may nevertheless develop despite preserved LVEF, particularly in patients with severe diastolic dysfunction and restrictive physiology [38,53]. Persistent NYHA class III–IV symptoms, worsening exercise capacity, pulmonary hypertension, or low-output physiology should prompt early evaluation at an advanced HF center. CPET provides objective assessment of functional limitation, while right-heart catheterization is useful when invasive hemodynamic characterization is required for transplant evaluation [2]. Heart transplantation remains the definitive therapy for selected patients with refractory advanced HF and should not be delayed [2,38,53]. Durable LV assist devices may serve as a bridge to transplantation in selected patients, particularly those with HCM-LVSD and adequate LV cavity size, although marked hypertrophy, restrictive filling, and small ventricular dimensions may increase technical and hemodynamic complexity [2,52]. Early recognition of advanced disease is therefore critical to avoid referral only after irreversible hemodynamic deterioration and irreversible pulmonary hypertension.

### 4.5. Lifestyle and Exercise

In recent years, recommendations regarding physical activity in HCM have shifted from broad restriction toward promotion of regular exercise and individualized exercise programs. Mild- to moderate-intensity recreational exercise is recommended because of its favorable effects on cardiorespiratory fitness, physical function, weight and general cardiovascular health [2,3]. In a randomized trial, 16 weeks of moderate-intensity exercise training produced a modest but significant improvement in peak VO_2_ without an excess of major adverse events or non-lethal arrhythmias [54]. The approach to vigorous exercise has also become less restrictive. In the prospective LIVE-HCM cohort, vigorous exercise was not associated with a higher incidence of death or life-threatening ventricular arrhythmias compared with moderate or lower-intensity activity [55]. Accordingly, universal restriction from vigorous recreational activity or competitive sport is no longer recommended [2]. Participation should instead follow individualized assessment of symptoms, SCD risk profile, LVOTO, ventricular function, prior syncope, and exercise-induced arrhythmias, with periodic reassessment in those undertaking high-intensity activity [2,3]. Genotype-positive/phenotype-negative individuals represent a particularly low-risk subgroup in whom competitive sport can generally be considered after appropriate evaluation [2,3]. Practical counseling in patients with obstructive HCM should include adequate hydration and avoidance of excessive alcohol intake, dehydration, and large meals, with smaller, more frequent meals favored to minimize hemodynamic fluctuations. Management should also address conventional cardiovascular risk factors, including obesity and hypertension

### 4.6. Pregnancy and Reproductive Counseling

Pregnancy is generally well tolerated in women with clinically stable HCM. Cardiovascular complications occur more frequently in those with pre-existing symptoms, ventricular dysfunction, significant LVOTO, or arrhythmic burden [2,56,57]. In the ROPAC registry, most pregnancies had favorable outcomes, but approximately one quarter were complicated by major cardiovascular events [57]. Pre-conception evaluation should assess symptoms, ventricular function, LVOTO, and arrhythmic risk and higher-risk patients should be managed with a multidisciplinary Pregnancy Heart Team [56]. Beta-blockers may be continued or initiated for symptomatic LVOTO or arrhythmias, with monitoring of fetal growth [2,56]. When beta-blockers are not tolerated, verapamil may be considered as an alternative, whereas diltiazem is not recommended during pregnancy [56]. Myosin inhibitors are not recommended during pregnancy, while other potentially fetotoxic cardiovascular therapies should be reviewed before conception [56]. Vaginal delivery is appropriate for most women, whereas severe HF, uncontrolled arrhythmias, or severe obstruction may require individualized delivery planning [56]. Pre-conception genetic counseling in sarcomeric HCM should address inheritance patterns, incomplete penetrance, variable expression, and reproductive options, including preimplantation genetic testing, prenatal diagnosis, and postnatal cascade testing [2,3]

## 5. Contemporary Management of Obstructive HCM

### 5.1. Conventional Pharmacological Therapy

Pharmacological treatment of symptomatic obstructive HCM has traditionally relied on negative inotropic and chronotropic agents aimed at reducing dynamic left ventricular outflow tract obstruction and improving symptoms. Non-vasodilating beta-blockers remain the recommended first-line treatment, while verapamil or diltiazem are considered when beta-blockers are ineffective or poorly tolerated [2,3]. Conventional therapies are symptom-directed, and convincing evidence about their ability to modify the natural history of HCM is lacking. Despite decades of clinical use, randomized evidence supporting beta-blockers has historically been limited. In a randomized, double-blind, placebo-controlled crossover trial of 29 patients with symptomatic obstructive HCM, metoprolol reduced LVOT gradients at rest and during and after exercise and improved NYHA class and patient-reported health status, without improving peak VO_2_ [58]. However, the small sample size and 2-week treatment duration preclude assessment of long-term benefits [58]. Verapamil and diltiazem are the principal alternatives to beta-blockers [2,3]. Their negative chronotropic and inotropic effects may improve symptoms and ventricular filling, although supporting evidence derives mainly from small historical studies. In a double-blind crossover study including 32 patients with HCM, oral diltiazem and verapamil showed broadly comparable symptomatic and functional effects [59]. Their vasodilating properties may nevertheless reduce systemic vascular resistance and aggravate obstruction in susceptible patients; verapamil should therefore be used cautiously in patients with hypotension, severe resting symptoms, or very high resting LVOT gradients [2]. Combination of beta-blockers with non-dihydropyridine calcium channel blockers is generally avoided because of the risk of excessive bradycardia and atrioventricular conduction disturbance. Disopyramide provides a stronger negative inotropic effect and remains an effective option in patients with persistent symptomatic LVOTO despite beta-blocker or calcium channel blocker therapy. However, disopyramide requires careful monitoring because of its potential proarrhythmic effects, including QT prolongation, and its frequent anticholinergic adverse effects, such as dry mouth, constipation, and urinary retention [2,3]. In a multicenter study of 118 patients with obstructive HCM, the drug reduced resting LVOT gradient and improved NYHA functional class, allowing a substantial proportion of patients to remain on medical treatment without proceeding to septal reduction therapy [60]. Long-term observational data support sustained efficacy of disopyramide in selected patients: among 92 patients treated for at least 5 years, resting and provoked LVOT gradients decreased by 37% and 57%, respectively, and approximately two-thirds remained on treatment after a median of 7.2 years [61]. A meta-analysis of 37 studies involving 1898 patients also reported substantial reductions in resting LVOT gradient with disopyramide [62]. However, heterogeneity across studies limits indirect comparisons between treatments, and gradient reduction alone does not establish improvements in functional capacity or long-term outcomes. Marked reductions in preload or afterload may worsen LVOTO; high-dose diuretics, vasodilators, and positive inotropic agents should therefore generally be avoided in symptomatic obstructive HCM, whereas low-dose diuretics may be used cautiously when congestion persists [2,3]. Persistent symptoms despite conventional therapy may warrant escalation to cardiac myosin inhibition or septal reduction therapy.

### 5.2. Cardiac Myosin Inhibitors

Cardiac myosin inhibitors (CMIs) represent the first pharmacological class specifically developed to target the sarcomeric hypercontractility underlying obstructive HCM. These small molecules modulate the cardiac myosin chemo-mechanical cycle. Mavacamten reduces steady-state cardiac myosin ATPase activity and slows phosphate release, thereby decreasing the proportion of myosin heads transitioning from weakly to strongly actin-bound, force-generating states and shifting the myosin population toward an energy-sparing super-relaxed state [63,64,65,66]. By reducing the number of actin-myosin cross-bridges available for force generation, CMIs attenuate hypercontractility and dynamic LVOTO without primarily relying on heart-rate reduction, while improving myocardial energetics and relaxation. In experimental HCM models, myosin inhibition also reduced hypertrophy, cardiomyocyte disarray, and myocardial fibrosis, providing a biological rationale for effects extending beyond acute negative inotropy [63]. However, CMIs should currently be regarded as mechanism-based rather than proven disease-modifying therapies, as an effect on heart failure progression, ventricular arrhythmias, or survival has not been established. Mavacamten provided the first phase III validation of this therapeutic strategy. In EXPLORER-HCM, 251 adults with symptomatic obstructive HCM and an LVOT gradient ≥50 mmHg were randomized to mavacamten or placebo for 30 weeks [67]. The primary composite functional endpoint was achieved in 37% of mavacamten-treated patients compared with 17% receiving placebo. Treatment also reduced post-exercise LVOT gradient by approximately 36 mmHg relative to placebo and improved peak oxygen consumption (pVO_2_), NYHA functional class, and health status [67]. VALOR-HCM extended these observations to patients with more advanced symptomatic obstruction who had already been referred for septal reduction therapy (SRT). Among 112 randomized patients, only 17.9% of those receiving mavacamten remained guideline-eligible for SRT or underwent the procedure at 16 weeks, compared with 76.8% receiving placebo [68]. Improvements in LVOTO, symptoms, and health status were maintained through 56 weeks. These findings demonstrated that pharmacological myosin inhibition can substantially modify the therapeutic pathway even in patients addressed to invasive treatment. Beyond hemodynamic and symptomatic improvement, imaging substudies suggested the possibility that myosin inhibition may favorably influence cardiac remodeling. Echocardiographic analysis of EXPLORER-HCM showed improvement in indices of diastolic function, left atrial volume, mitral regurgitation, and other structural and functional parameters [69], while the CMR substudy demonstrated reductions in LV mass index and maximal wall thickness [70]. These observations support biological effects beyond instantaneous gradient reduction, but their long-term clinical significance remains uncertain. Aficamten subsequently provided independent validation of cardiac myosin as a therapeutic target. In the phase II REDWOOD-HCM trial, the drug produced rapid and substantial reductions in resting and provoked LVOT gradients, with complete hemodynamic response, defined as a resting gradient <30 mmHg and Valsalva gradient <50 mmHg, achieved in 79% and 93% of patients in the two active-treatment cohorts compared with 8% receiving placebo [71]. The phase III SEQUOIA-HCM trial then randomized 282 symptomatic patients to aficamten or placebo for 24 weeks [72]. Mean pVO_2_ increased by 1.8 mL/kg/min with aficamten and remained essentially unchanged with placebo, corresponding to a between-group difference of 1.7 mL/kg/min. All ten hierarchically tested secondary endpoints also favored aficamten, including NYHA functional class, KCCQ clinical summary score, Valsalva LVOT gradient, exercise workload, and eligibility for SRT [72]. An imaging substudy of SEQUOIA-HCM demonstrated reductions in LV mass index, maximal wall thickness, left atrial volume index, and native T1 after 24 weeks of aficamten treatment, without significant changes in LGE burden or extracellular volume fraction [73]. The MAPLE-HCM trial moved the field beyond placebo-controlled add-on therapy by directly comparing targeted myosin inhibition with conventional first-line treatment. In this double-blind trial, 175 patients with symptomatic obstructive HCM were randomized to aficamten or metoprolol monotherapy for 24 weeks [74]. PVO_2_ increased by 1.1 mL/kg/min with aficamten and decreased by 1.2 mL/kg/min with metoprolol, yielding a between-group difference of 2.3 mL/kg/min. Aficamten was also associated with greater improvements in LVOTO, NYHA functional class, KCCQ-CSS, and NT-proBNP levels [74]. Current European and American guidelines recommend beta-blockers or CCBs as initial therapy [2,3] and CMIs should be started if these two drug categories show poor efficacy. As previously stated, even though these drugs have been used in clinical practice for decades, and their safety profiles are well established, their efficacy in LVOTO reduction is still debated. MAPLE-HCM firstly compared a CMI and a traditional drug showing a better reduction in LVOTO reduction together with VO_2_ improvement in aficamten arm VS a VO_2_ reduction in metoprolol arm. This last data highlights a common finding in patients performing CPET under BB treatment: BB-induced chronotropic incompetence possibly worsening patients’ symptoms and exercise capacity, often limiting BB titration and reducing the (possible) LVOTO reduction ability.

Taken together, this evidence might theoretically support the use of CMIs earlier in the treatment of symptomatic obstructive HCM, even though CMI monotherapy data are still lacking and long-term efficacy and safety data are still accumulating. In the long-term extension of EXPLORER-HCM, mavacamten maintained reductions in resting and Valsalva LVOT gradients and improvements in functional class during follow-up extending beyond three years [75]. Similarly, prolonged aficamten treatment in FOREST-HCM was associated with sustained hemodynamic and symptomatic improvement [76]. These studies support durability of pharmacological efficacy, but their open-label design and limited duration relative to the lifetime course of HCM preclude conclusions regarding prevention of HF progression, ventricular arrhythmias, or mortality. Excessive myosin inhibition may cause LV systolic dysfunction, which has generally been reversible after dose adjustment or treatment interruption in clinical trials [67,72,75]. Accordingly, CMI therapy requires serial echocardiographic monitoring of LVEF, with dose adjustment or treatment interruption according to agent-specific prescribing requirements [2,3]. Pharmacokinetic profiles differ between the two agents: mavacamten has a half-life of 6–9 days, prolonged to approximately 23 days in CYP2C19 poor metabolizers, and is metabolized mainly by CYP2C19, with contributions from CYP3A4 and CYP2C9. Aficamten has a shorter half-life of approximately 3.4 days. These differences influence drug–drug interactions, dose titration, and treatment interruption, without implying comparative clinical superiority [66,72]. The interaction between CMIs and conventional negative inotropic therapy is also being defined. In patients receiving aficamten in combination with disopyramide, concomitant administration was generally well tolerated, while subsequent withdrawal of disopyramide did not appear to compromise the hemodynamic or symptomatic response to aficamten [77]. These findings suggest that prolonged exposure to multiple negative inotropic agents may not be necessary in all patients once effective myosin inhibition has been established, although the available evidence remains limited. Finally, candidacy for CMI therapy should not be regarded as a fixed consequence of a single gradient or clinical assessment. Real-world studies have shown that the population potentially eligible for myosin inhibition differs substantially from that enrolled in pivotal trials and that only a proportion of patients with obstructive HCM and persistent LVOTO ultimately fulfill treatment criteria. In a large multicenter Italian cohort, approximately half of patients with persistent obstruction were eligible for mavacamten according to EXPLORER-HCM or European guideline criteria [78]. Similarly, analysis of the French REMY registry showed that eligibility may change longitudinally as symptoms, LVOTO, ventricular function, and response to conventional therapy evolve [79]. Some initially eligible patients may improve after optimization of conventional treatment, whereas previously asymptomatic patients may subsequently become candidates for therapy. These observations reinforce the importance of repeated clinical assessment when considering CMI initiation.

### 5.3. Septal Reduction Therapy

Septal reduction therapy (SRT) remains an established treatment for patients with obstructive HCM who have persistent symptoms attributable to LVOTO despite medical therapy. Candidates generally have severe functional limitation, usually NYHA class III–IV or occasionally exertional syncope or other relevant symptoms, together with a resting or physiologically provoked LVOT gradient ≥50 mmHg [2,3]. Selection should not rely on gradient magnitude alone but should integrate the mechanism of obstruction, septal morphology, mitral and subvalvular anatomy, concomitant cardiac disease, comorbidities, procedural risk, and patient preference within an experienced HCM team. Surgical septal myectomy remains the reference invasive treatment and is particularly suited to patients with complex obstructive anatomy. Contemporary surgery has evolved beyond the original limited Morrow resection toward a more individualized approach in which extended septal resection may be combined with procedures directed at abnormal papillary muscles, chordae, or mitral-valve structures when these contribute to systolic anterior motion and obstruction [80]. Myectomy also permits concomitant treatment of other surgical disease, including primary mitral pathology, coronary artery disease, or atrial fibrillation. Alcohol septal ablation (ASA) is the principal percutaneous alternative. Selective alcohol injection into an appropriate septal perforator creates a controlled basal septal infarction followed by thinning and remodeling of the obstructing myocardium. ASA avoids sternotomy and cardiopulmonary bypass and is therefore particularly attractive in older patients or those with relevant comorbidities or increased surgical risk [2,3]. However, it depends on suitable septal coronary anatomy and cannot directly correct abnormalities of the mitral valve, chordae, or papillary muscles. Compared with myectomy, observational evidence suggests a higher risk of atrioventricular block requiring permanent pacing, greater residual obstruction, and more frequent repeat septal reduction procedures [3]. Contemporary outcomes of SRT are favorable when procedures are performed in experienced centers. In the international SHaRe registry, 1832 patients underwent SRT, approximately three-quarters by myectomy and one-quarter by ASA; 30-day mortality was 0.4%, with low long-term mortality and a higher need for repeat intervention after ASA [81]. Procedural expertise remains critical, with nationwide data demonstrating a clear volume-outcome relationship for septal myectomy [82]. Current guidelines place SRT among the established treatment options for symptomatic obstructive HCM, with shared decision-making increasingly relevant in the era of CMIs [2,3]. Treatment selection should consider patient preference for potentially lifelong pharmacological therapy versus an invasive procedure offering durable relief of LVOTO, alongside procedural risk and anatomical suitability. Surgically modifiable features, such as intrinsic mitral-valve disease, papillary muscle displacement, and marked septal hypertrophy, may further favor surgical approach.

## 6. VI Contemporary Management of Non-Obstructive HCM

### 6.1. Conventional Symptom-Directed Therapy

In patients with non-obstructive HCM and preserved LVEF, beta-blockers or non-dihydropyridine calcium channel blockers remain the conventional treatment for exertional dyspnea or angina [2,3]. Their rationale is based on heart-rate reduction, prolonged diastolic filling, and reduced myocardial oxygen demand, although supporting evidence is limited and no conventional agent has convincingly modified disease progression. Low-dose diuretics may be used when elevated filling pressures or congestion contribute to symptoms, while excessive volume depletion should be avoided in patients with small, poorly compliant ventricles [2,3]. Renin-angiotensin system inhibitors (RASi) may be indicated for concomitant hypertension or other conventional indications but have not demonstrated HCM-specific benefit in established disease. In the randomized INHERIT trial, losartan did not reduce LV mass or myocardial fibrosis and did not improve functional class compared with placebo [83]. In highly selected patients with severe apical or midventricular hypertrophy, a small LV cavity, and refractory symptoms, apical myectomy may be considered [84]. Evidence supporting SGLT2 inhibitor use specifically in non-obstructive HCM remains insufficient to establish their role in routine treatment.

### 6.2. Cardiac Myosin Inhibition in Non-Obstructive HCM

The rationale for cardiac myosin inhibition in non-obstructive HCM differs from that in obstructive disease, as treatment cannot rely on relief of LVOTO and any benefit must instead derive from effects on myocardial contractility, diastolic function, and energetics. MAVERICK-HCM provided initial proof of biological activity: in 59 symptomatic patients, mavacamten reduced NT-proBNP and cardiac troponin I, although the phase II study was primarily designed to assess safety and was not powered for clinical efficacy [85]. The subsequent phase III ODYSSEY-HCM trial randomized 580 patients with symptomatic non-obstructive HCM to mavacamten or placebo for 48 weeks [86]. Neither co-primary endpoint was significantly improved: the placebo-corrected change in pVO_2_ was 0.47 mL/kg/min (*p* = 0.07), while the difference in KCCQ Clinical Summary Score was 2.7 points (*p* = 0.06). Reductions in LVEF and treatment interruptions were also more frequent with mavacamten. Thus, despite evidence of pharmacological activity, mavacamten did not significantly improve exercise capacity or patient-reported symptoms in this population [86]. Aficamten showed preliminary signals of efficacy in REDWOOD-HCM Cohort 4, an open-label study of 41 symptomatic patients, with improvements in NYHA functional class and KCCQ-CSS and reductions in NT-proBNP and high-sensitivity cardiac troponin I [87]. These findings led to ACACIA-HCM, a phase III trial enrolling 517 patients with symptomatic non-obstructive HCM [88]. At 36 weeks, aficamten significantly improved both primary endpoints: the placebo-corrected increase in pVO_2_ was 0.67 mL/kg/min (*p* = 0.003), while KCCQ-CSS improved by an additional 3.0 points compared with placebo (*p* = 0.02). LVEF <50% occurred in 10.5% of aficamten-treated patients versus 0.8% receiving placebo [88]. It was reversible with treatment interruption or adjustment [88]. This trial showed, for the first time, the potential benefit of aficamten in HCM in terms of functional capacity and quality of life amelioration; however, the data regarding LVEF reduction raised some concerns. Among these 27 patients showing LVEF reduction <50%, 21 reduced temporarily or permanently reduced the drug dose but continued the treatment and only 3% discontinued the drug [88]. Safety does not seem to be a real concern, though, but both efficacy in the real world and the rate of suspension should be evaluated in larger numbers.

Although ODYSSEY-HCM and ACACIA-HCM yielded contrasting results, these findings do not establish different biological effects of mavacamten and aficamten, as trial design, patient populations, treatment exposure, and dose titration differed, and direct comparative evidence is lacking. Overall, these findings support a potential clinical benefit of myosin inhibition in non-obstructive HCM, while further studies are needed to identify predictors of response and establish whether improvements translate into sustained benefit and reduced heart failure progression. Table 1.

### 6.3. Emerging Pharmacological Strategies

Beyond cardiac myosin inhibition, myocardial energetics represents another potential therapeutic target in non-obstructive HCM. Earlier attempts at metabolic modulation were disappointing: in a randomized trial of 51 patients, trimetazidine failed to improve exercise capacity and was associated with lower peak oxygen uptake compared with placebo [89]. More recently, ninerafaxstat, a cardiac mitotrope designed to improve myocardial energetic efficiency by shifting substrate utilization toward glucose oxidation, has renewed interest in this approach. In the phase II IMPROVE-HCM trial, 67 patients with symptomatic non-obstructive HCM and impaired exercise capacity were randomized to ninerafaxstat or placebo for 12 weeks [90]. Ninerafaxstat significantly improved ventilatory efficiency, with a placebo-corrected reduction in the VE/VCO_2_ slope of 2.1 units, although pVO_2_ was not significantly changed. KCCQ-CSS improved numerically in the overall population, with a significant benefit in a post hoc subgroup of patients with greater baseline symptomatic impairment [90]. These findings support myocardial energetics as a potentially relevant therapeutic target but require confirmation in larger studies. Ninerafaxstat is currently being evaluated in the randomized phase IIb FORTITUDE-HCM trial in symptomatic non-obstructive HCM [91]. A complementary strategy is being investigated with sotagliflozin, a dual SGLT1/2 inhibitor. SONATA-HCM is a phase III randomized trial evaluating sotagliflozin in approximately 500 patients with symptomatic HCM, including both obstructive and non-obstructive phenotypes, with change in KCCQ Clinical Summary Score as the primary endpoint [92]. Whether the established benefits of SGLT inhibition in heart failure translate specifically to sarcomeric HCM remains uncertain, as currently available HCM data are largely observational and frequently derived from metabolically complex populations. Together, these programs broaden the therapeutic landscape beyond current strategies and illustrate the potential for targeting distinct mechanisms contributing to exercise limitation and symptoms in non-obstructive HCM. As with CMIs, identifying the physiological phenotype most likely to respond may ultimately be as important as the therapeutic target itself.

## 7. Emerging Disease-Modifying and Precision Strategies

### 7.1. Earlier Intervention

The VANISH trial provided the principal clinical proof of concept for this strategy. In this phase II randomized trial, 178 patients aged 8–45 years with early-stage sarcomeric HCM received valsartan or placebo for 2 years [93]. Valsartan favorably affected a prespecified multidimensional endpoint integrating cardiac structure, function, and biomarkers. Notably, these findings contrast with the neutral results of RASi treatment in INHERIT [83], suggesting that disease stage may play a role in therapeutic response. VANISH was not powered for major clinical outcomes, and whether early treatment prevents heart failure, arrhythmias, or other HCM-related complications remains unknown [93]. Nevertheless, it introduced an important conceptual shift: future disease-modifying strategies may need to target patients before advanced disease development. This principle becomes particularly relevant as therapies are moving forward to underlying molecular defects.

### 7.2. Gene-Based Therapies

Gene-based therapy represents the most upstream potential disease-modifying strategy for genetically defined HCM, aiming to correct or compensate for the primary molecular defect rather than its phenotypic consequences. The appropriate strategy depends on the underlying pathogenic mechanism. Gene replacement may restore functional protein levels in HCM caused by haploinsufficiency, whereas dominant-negative variants may require suppression or correction of the mutant allele to prevent interference from the abnormal protein. *MYBPC3* currently represents the most advanced therapeutic target. Truncating pathogenic variants commonly lead to reduced cardiac myosin-binding protein C (MYBPC), providing a direct rationale for restoration of protein expression. In preclinical models, adeno-associated virus serotype 9 (AAV9)-mediated *MYBPC3* replacement increased myocardial MyBP-C abundance, attenuated hypertrophy, improved cardiac function, and prolonged survival [94]. TN-201, an investigational gene-replacement therapy for *MYBPC3*-associated HCM, uses an AAV9 vector to deliver a functional *MYBPC3* transgene to cardiomyocytes. Initial results from the phase Ib/II MyPEAK-1 study included six symptomatic adults with pathogenic or likely pathogenic truncating *MYBPC3* variants treated across two dose cohorts [95]. Myocardial biopsies demonstrated cardiomyocyte transduction, detection of vector-derived *MYBPC3* expression, and increased MyBP-C abundance, accompanied by preliminary favorable changes in cardiac biomarkers and measures of LV hypertrophy. Treatment was generally well tolerated in this very small initial cohort, although vector-associated immune responses and reversible elevations in liver enzymes required specific clinical management [95]. These findings are first-in-human proof of mechanism and should not be considered evidence of clinical efficacy. Other gene-directed technologies remain preclinical. Adenine base editing offers a strategy to correct pathogenic point mutations without inducing double-strand DNA breaks. In patient-derived cardiomyocytes and humanized mouse models, this approach restored the normal MYH7 sequence by correcting the p.R403Q variant, with favorable effects on cellular function and prevention of the HCM phenotype in treated mice [96]. A separate preclinical study evaluated both correction of the same variant through base editing and selective Cas9-mediated disruption of the mutant allele. The latter approach aims to suppress production of the abnormal β-myosin protein while preserving expression from the unaffected allele. Both strategies prevented HCM development in experimental models, although selective allele disruption was associated with dose-dependent toxicity [97]. Unlike conventional pharmacotherapy, these approaches may produce potentially permanent molecular effects. This distinction raises important challenges for delivery efficiency, genomic specificity, and long-term safety. AAV-based therapy requires effective cardiomyocyte transduction while limiting systemic toxicity and immune responses; pre-existing neutralizing antibodies may restrict eligibility, and limited opportunities for redosing increase the importance of the initial treatment strategy. Genome editing introduces additional concerns regarding off-target alterations and permanent DNA modification. Clinical implementation will therefore require careful molecular and immunological selection, specialized treatment pathways, and prolonged surveillance. The optimal therapeutic window also remains uncertain: intervention after extensive fibrosis and adverse remodeling may have limited potential to reverse established disease, whereas treatment of minimally affected or phenotype-negative variant carriers would require an exceptionally favorable long-term safety profile and reliable prediction of disease progression. Gene-based therapy may ultimately make genotype an active determinant of treatment selection, but its clinical role will depend on molecular mechanism, phenotype, disease stage, and technology-specific safety. Even though gene editing approaches seem promising, they have to prove their efficacy and sustainability in clinical practice.

## 8. Future Directions and Conclusions

Hypertrophic cardiomyopathy remains a complex and multifaceted disease in which careful clinical assessment and detailed characterization of cardiovascular physiology are fundamental to appropriate management. Symptoms, haemodynamics, arrhythmic risk, ventricular function, myocardial fibrosis, exercise limitation, and mechanisms of obstruction may vary substantially between patients and over time. For this reason, management cannot rely on a single parameter or therapeutic algorithm, but requires integration of clinical evaluation, electrocardiography, multimodality imaging, functional assessment, and longitudinal reassessment [2,3]. Cardiac myosin inhibitors have substantially changed the therapeutic landscape by introducing the first mechanism-based pharmacological therapies capable of directly modulating sarcomeric contractility. In obstructive HCM, they have expanded treatment beyond conventional negative inotropic therapy and provided a pharmacological alternative to septal reduction in appropriately selected patients [67,72,74]. However, septal reduction therapy remains a highly effective and durable strategy, particularly when obstruction is driven by complex septal, mitral, or subvalvular anatomy. A major contemporary challenge is therefore to define more precisely which patients are best treated pharmacologically and which should be referred for definitive mechanical correction [80,81]. In non-obstructive HCM, recent trials have further emphasized the heterogeneity of the disease and the importance of identifying the physiological substrate responsible for symptoms. The divergent results observed across cardiac myosin inhibitor studies suggest that therapeutic response is unlikely to be uniform and that appropriate patient selection will be central to future treatment strategies [86,88]. Finally, gene-based therapies may ultimately produce the most profound change in HCM management by moving treatment from modulation of downstream physiology toward correction of the underlying molecular defect. Early human experience with *MYBPC3* gene replacement has demonstrated biological feasibility, although efficacy, durability, safety, and optimal timing remain to be established [95]. HCM management may therefore progressively evolve toward an increasingly integrated model in which clinical phenotype, cardiovascular physiology, imaging, genotype, and disease stage collectively determine both treatment selection and timing.

## Figures and Tables

**Table 1:** Main cardiac myosin inhibitor trials in hypertrophic cardiomyopathy.

Trial/Drug	Population	Duration	Primary Endpoint	Main Results	Safety
Obstructive HCM					
EXPLORER-HCM Mavacamten	oHCM; *n* = 251	30 wk	pVO_2_/NYHA response	37% vs. 17% response	LVEF <50%: 5.7% vs. 1.6%
VALOR-HCM Mavacamten	oHCM; SRT candidates; *n* = 112	16 wk	SRT eligibility/proceeding	17.9% vs. 76.8%	LVEF-related treatment holds
REDWOOD-HCM Aficamten	oHCM; phase II	10 wk	Safety; LVOT response	LVOT gradients decreased	Reversible LVEF decrease
SEQUOIA-HCM Aficamten	oHCM; *n* = 282	24 wk	Change in pVO_2_	+1.7 mL/kg/min	LVEF <50%: 3.5% vs. 0.7%
MAPLE-HCM Aficamten	oHCM; *n* = 175; vs. metoprolol	24 wk	Change in pVO_2_	+2.3 mL/kg/min	Similar AE rates
Non-obstructive HCM					
MAVERICK-HCM Mavacamten	nHCM; *n* = 59	16 wk	Safety/tolerability	NT-proBNP, cTnI decreased	Reversible LVEF decrease
ODYSSEY-HCM Mavacamten	nHCM; *n* = 580	48 wk	pVO_2_; KCCQ-CSS	+0.47 mL/kg/min; +2.7 pts (NS)	LVEF <50%: 21.5% vs. 1.7%
REDWOOD-HCM C4 Aficamten	nHCM; *n* = 41	10 wk	Safety/tolerability	NYHA, biomarkers improved	LVEF decrease reported
ACACIA-HCM Aficamten	nHCM; *n* = 517	36 wk	pVO_2_; KCCQ-CSS	+0.67 mL/kg/min; +3.0 pts	LVEF <50%: 10.5% vs. 0.8%

Abbreviations: AE, adverse event; cTnI, cardiac troponin I; HCM, hypertrophic cardiomyopathy; KCCQ-CSS, Kansas City Cardiomyopathy Questionnaire Clinical Summary Score; LVEF, left ventricular ejection fraction; LVOT, left ventricular outflow tract; NS, nonsignificant; NYHA, New York Heart Association; pVO_2_, peak oxygen uptake; SRT, septal reduction therapy.

## Data Availability

No new data were created or analyzed in this study. Data sharing is not applicable to this article.
